# Zearalenone Promotes Cell Proliferation or Causes Cell Death?

**DOI:** 10.3390/toxins10050184

**Published:** 2018-05-02

**Authors:** Wanglong Zheng, Bingjie Wang, Xi Li, Tao Wang, Hui Zou, Jianhong Gu, Yan Yuan, Xuezhong Liu, Jianfa Bai, Jianchun Bian, Zongping Liu

**Affiliations:** 1 College of Veterinary Medicine, Yangzhou University, Yangzhou 225009, China; zhengwanglong@163.com (W.Z.); wangbingjie123188@163.com (B.W.); Lixiorqian@sina.com (X.L.); wtao6550@yzu.edu.cn (T.W.); zouhui@yzu.edu.cn (H.Z.); jhgu@yzu.edu.cn (J.G.); yuanyan@yzu.edu.cn (Y.Y.); liuxuezhong68@163.com (X.L.); 2 Jiangsu Co-Innovation Center for Prevention and Control of Important Animal Infectious Diseases and Zoonoses, Yangzhou 225009, China; 3 Joint International Research Laboratory of Agriculture and Agri-Product Safety, The Ministry of Education of China, Yangzhou University, Yangzhou 225009, China; 4 Kansas State Veterinary Diagnostic Laboratory, Kansas State University, 1800, Denison Avenue, Manhattan, KS 66506, USA; jbai@vet.k-state.edu

**Keywords:** zearalenone, cell proliferation, cell death, estrogen-like effects, apoptosis

## Abstract

Zearalenone (ZEA), one of the mycotoxins, exerts different mechanisms of toxicity in different cell types at different doses. It can not only stimulate cell proliferation but also inhibit cell viability, induce cell apoptosis, and cause cell death. Thus, the objective of this review is to summarize the available mechanisms and current evidence of what is known about the cell proliferation or cell death induced by ZEA. An increasing number of studies have suggested that ZEA promoted cell proliferation attributing to its estrogen-like effects and carcinogenic properties. What’s more, many studies have indicated that ZEA caused cell death via affecting the distribution of the cell cycle, stimulating oxidative stress and inducing apoptosis. In addition, several studies have revealed that autophagy and some antioxidants can reverse the damage or cell death induced by ZEA. This review thoroughly summarized the metabolic process of ZEA and the molecular mechanisms of ZEA stimulating cell proliferation and cell death. It concluded that a low dose of ZEA can exert estrogen-like effects and carcinogenic properties, which can stimulate the proliferation of cells. While, in addition, a high dose of ZEA can cause cell death through inducing cell cycle arrest, oxidative stress, DNA damage, mitochondrial damage, and apoptosis.

## 1. Introduction

Zearalenone (ZEA), one of the mycotoxins, mainly comes from the feed which was contaminated by some Fusarium and Gibberella species in the field and farm or in the period and storage [1,2]. Although before harvest time, the cereals infected by Fusarium may accumulate ZEA in the field, numerous evidence has revealed that a high level of ZEA could be naturally occurring in the corn-based animal feeds, and thus be attributed to the improper storage methods rather than occurring in the field [3,4]. The trade of these contaminated cereal commodities may contribute to the worldwide dispersal of ZEA [5]. Several studies have shown that ZEA exerted different mechanisms of toxicity in different cell types at different doses. ZEA and its derivatives can not only stimulate the cell growth but also inhibit the cell viability and cause cell death including apoptosis and necrosis [6,7,8,9].

Recently accumulating evidence has shown showed that ZEA can stimulate cell proliferation in different cells. ZEA showed a powerful activity to stimulate cell proliferation starting at 10^−10^ M to a maximum at 10^−8^ M [10]. ZEA could stimulate T47D cells growth and, compared with control cells, the stimulating effect was 2-fold in 10^−8^ M group [11]. What’s more, several studies have indicated that the derivatives of ZEA can also stimulate cell growth. α-zearalanol (α-ZAL), one of the derivatives of ZEA, could effectively stimulate the proliferation of BMS cells, induce differentiation into osteoblasts and suppress osteoclastogenesis formation [12]. α-Zearalenol (α-ZEL), the another one derivative of ZEA, showed a strong effect of stimulating on granulosa cells, even when treated with fumonisin B1 (FB1) which could inhibit the growth of granulosa cells [13]. In addition, studies have suggested that ZEA could increase the expressions of cell cycle-regulated proteins such as Cdk4 and cyclin D1 in TM3 cells [8].

However, a lot of other studies have revealed that ZEA can inhibit the cell viability and cause cell death including apoptosis and necrosis. After treatment with ZEA (15–60 μM) for 24 h, the viability of Sertoli cells was decreased markedly [14]. After treatment with ZEA (3–300 μM) could cause a significantly decrease in cell viability, and the IC50 values for ZEA was 80 μM [15]. ZEA could cause cell necrosis and apoptosis in the RAW264.7 cells and in the early stages, the main cytotoxicity was causing necrosis [16]. ZEA caused similar necrotic profiles in both resting and stimulated human peripheral blood mononuclear cells in vitro [17]. The study from porcine granulosa cells have suggested that ZEA caused necrosis through mitochondrial pathway mediated by caspase-3 and caspase-9 [18]. What’s more, study indicated that ZEA can affect the expressions of cell cycle regulated proteins including Cyclin-B1, CyclinD1, CDK2 and CDK4 and affect the cell cycle distribution, which might cause the decrease in the cell viability [19]. In addition, many studies have revealed that ZEA could cause cell apoptosis and necrosis. ZEA induced obvious apoptosis in endometrial stromal cells (ESCs), PK15 cells, Leydig cells, Sertoli cells, raw 264.7 macrophages and porcine granulosa cells [18,20,21,22,23].

In the face of complicated and opposite conclusions that ZEA could not only stimulate cell proliferation but also cause cell death, several crucial and meaningful questions naturally arise: when does ZEA promote cell proliferation? When does ZEA cause cell death? How does ZEA stimulate the cell growth? How does ZEA induce cell death? What medicines can protect the cytotoxicity of ZEA? Thus, the purpose of this article is to discuss and summarize the available mechanisms and current data of what is known about the cell proliferation or cell death induced by ZEA.

## 2. The Metabolic Process of ZEA

**Figure 1:**
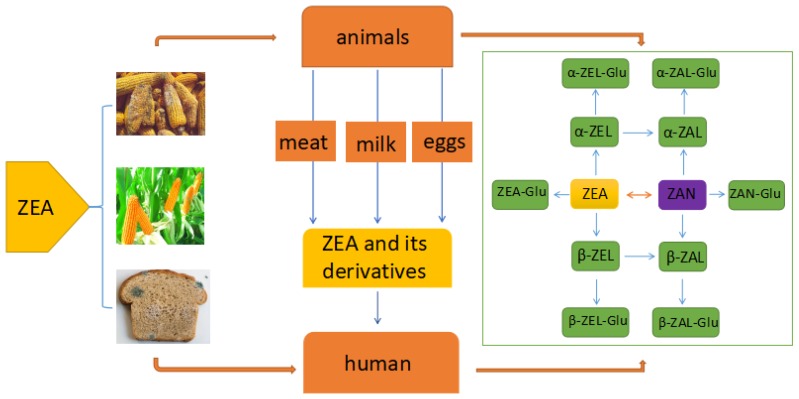
The major biotransformation pathways of ZEA Abbreviations: Zearalenone (ZEA); α-zearalenol (α-ZEL); β-zearalenol (β-ZEL); Zearalanone (ZAN); α-zearalanol (α-ZAL); β-zearalanol (β-ZAL); ZEA-Glucuronide (ZEA-Glu); α-zearalenol-Glucuronide (α-ZEL-Glu); β-zearalenol-Glucuronide (β-ZEL-Glu); Zearalanone-Glucuronide (ZAN-Glu); α-zearaanol-Glucuronide (α-ZAL-Glu); β-zearalanol-Glucuronide (β-ZAL-Glu).

The main way for human and animals exposure to ZEA is consuming the cereal grains and derived products (Figure 1) which may be contaminated by toxigenic fungi species of Fusarium in field or during food production, processing and storage [24]. These toxigenic fungi are considered as significantly harmful pathogens due to producing mycotoxin in the safety and quality of cereal grains [25]. Except the cereal grains and derived products, the intake of ZEA and its derivatives can be occurred via consuming the animal-origin food such as eggs, milk, and meat which were derived from the animals that were exposed to ZEA or were injected its derivatives for stimulating growth [26,27]. The average concentration of ZEA in bovine milk was estimated in 0.125 µg/L and the concentration of ZEA in the dairy cattle diets was estimated in 194.9 µg/Kg [28]. A rapid and sensitive method UHPLC–MS/MS which use electrospray ionization (ESI) in multiple reaction monitoring mode (MRM) has been developed for detecting the ZEA, and its derivatives simultaneously in different types of milk including milk powder, liquid milk and raw milk. [29].

ZEA was rapidly absorbed following oral intake during subsequent metabolism mainly in the liver and intestine. It was transformed into α-zearalenol (α-ZEA), β-zearalenol (β-ZEA), zearalanone (ZAN), α-zearalanol (α-ZAL) and β-zearalanol (β-ZAL) and all of which were subsequently conjugated to glucuronic acid [30]. The metabolism of ZEA can be divided into two phases including Phase-I metabolism and Phase-II metabolism. At the phase-I, the ketone group in ZEA or ZAN which is a semi-synthetic mycoestrogen and a derivative of ZEA was reduced by aliphatic hydroxylation to metabolize the corresponding alcohol. ZEA was converted to α-ZEL and β-ZEL and ZAN was converted to α-ZAL and β-ZAL, which was catalyzed by 3α -hydroxysteroid dehydrogenase (3α-HSD) or 3β–hydroxysteroid dehydrogenase (3β -HSD) [31]. At the phase- II the metabolites from phase-I were glucuronidated and sulfated. The glucuronic acid group was supplied by uridine 5′-diphosphate glucuronic acid (UDPGA) which was catalyzed by uridine 5′-diphosphate glucuronosyltransferase (UGT) [31]. Except the liver and intestine, a lot of other tissues such as the prostate, testis, kidney, hypothalamus and ovary also contain 3α-HSD and 3β-HSD and have the ability to metabolize ZEA [32]. What’s more, study also suggested that enterohepatic cycling and biliary excretion are important processes in the metabolizing of ZEA. The glucuronide of ZEA was substantially excreted in the bile to be re-absorbed and metabolized further by intestinal mucosal cells, ultimately entering the liver and the systemic circulation via the portal blood supply [30,33]. In addition, studies have suggested that the enterohepatic cycling causes the prolonged retention of ZEA and these derivatives in the circulatory system, retarding its elimination and enhancing the duration of adverse effects [32]. 

## 3. The Molecular Mechanisms of ZEA Promoted the Cell Proliferation

### 3.1. ZEA and Its Derivatives Can Exert the Estrogen-Like Effects Which Can Stimulate the Cell Proliferation

**Figure 2:**
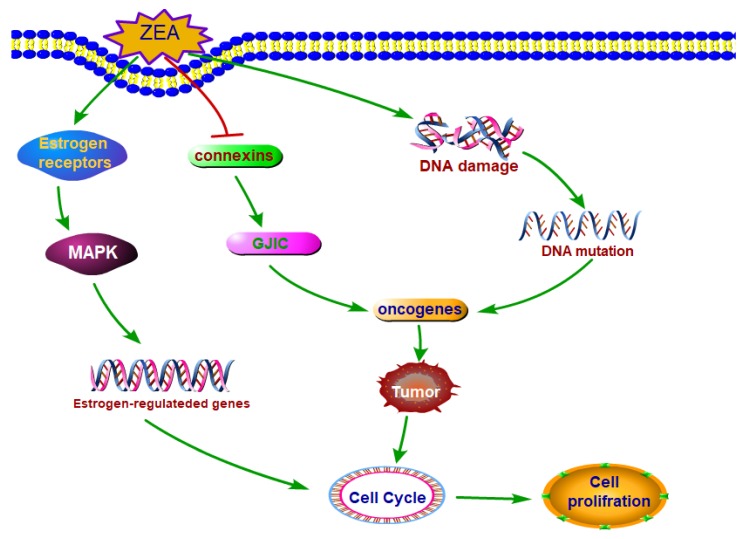
The main molecule mechanisms of ZEA stimulated cell proliferation. ZEA and its derivatives can exert the estrogen-like effects which can bind to estrogenic receptors and stimulate the cell proliferation. ZEA can induce tumor through damaging DNA and disturbing the function of GJIC. Abbreviations: Zearalenone (ZEA); mitogen-activated protein kinase (MAPK); gap junctional intercellular communication (GJIC).

ZEA and its metabolites have structural analogy to estrogen (Figure 2), thus they can bind to estrogen receptors (ERs) and exert the estrogen-like effects [34]. In vitro, ZEA and its metabolites were flexible enough to bind mammalian estrogenic receptors in human cancer MCF-7 cells at dose ranging from 6.25 to 25 µM [34]. In vivo, ZEA caused multiple estrogenic toxic actions including interfering the processes of establishing and maintaining pregnancy, such as the decidual response, the embryo migration from oviducts to uteri and the activation of luteal function after giving a daily injections of ZEA (2, 4, and 8 mg/kg) during the pregnancy period in female mice [35]. What’s more, many studies have indicated that ZEA, β-ZAL and β-ZEL could be classified as partial agonists, while α-ZEA and α-ZAL could be classified as total agonists. The estrogenic activities are different and can be ranked as follows: α-ZEL = α-ZAL > ZAN > ZEA = β-ZAL > β-ZEL in human breast cancer cells in vitro [36]. The reason why α-ZEL showed a stronger estrogenic activity than ZEA and other derivatives is that α-ZEL does not bind to the carrier protein which can increase its ability to bind other receptors such as estrogenic receptors [34]. An increasing number of studies have suggested that steroid hormones have important regulatory roles in cell proliferation, and estrogen can stimulate different cells growth [37,38]. Thus, one of the reasons that ZEA can stimulate the cell proliferation can be attributed to its estrogen-like effects.

Estrogen regulates the biological processes through estrogen receptors which can activate cell signaling pathways and the extracellular-signal-regulated kinase 1/2 (ERK1/2) mediates the estrogen-like signal for cell proliferation [35,38]. Studies have indicated that17β-estradiol promoted pig Sertoli cells proliferation through estrogen receptor to activate cAMP and ERK1/2 [39]. ZEA also can rapidly trigger the activation of the ERK1/2 signal way in the MCF-7 cells, which is similar to the response of the MCF-7 cells exposure to E2 [36]. Studies have showed that some estrogen-like compounds including ginsenoside Rg1, genistein or equol mediated the cell proliferation of MCF0-7 cells also through the ERK1/2 signal pathway [40]. Although, the underlying mechanism of activating ERK1/2 is also unclear, accumulating evidence has suggested that membrane-bound classic estrogen receptor α and estrogen receptor β and G-protein-coupled protein, GPR30, participated in the process of estrogen-induced signaling [41,42].

What’s more, ZEA stimulated cell proliferation through changing the expression of estrogen-responsive genes. ZEA can affect the expression of estrogen-regulated genes which can regulate physiological estrogen responses including mediating the proliferation of cells due to the ability of binding ERs [43]. Studies have also shown that exposures the MCF-7 cells to ZEAand E2, ZEA and E2 had similar expression profiles of estrogen-responsive genes [36]. ZEA can stimulate the expression of CXCL12 gene which was suggested to be linked to the proliferation of the MCF-7 cells [10]. The study from pig spleen indicated that after treatment with ZEA 14% of total genes related to proliferation were altered. The expressions of JAG1 and TGFβ-2 genes were increased and the FoxP3 and TLR7 were decreased significantly [44]. In addition, except the genomic signaling, estrogen receptors can also stimulate through non-genomic signaling events in a slower time course.

### 3.2. ZEA Exhibited the Carcinogenic Property

Several studies have shown that ZEA has the carcinogenic property which may be the other one reason for stimulating the cells proliferation. A study has shown that ZEA could promote HCT116 cells proliferation and also promote cell migration and colony formation which are the hallmarks of carcinogenic property [45]. ZEA-induced DNA lesions may cause DNA fragmentation and disturb the progression of cell cycle in indifferent cells including Vero, Caco-2 and DOK [46]. A study has revealed that α-ZAL could exert a biphasic behavior and exert beneficial or harmful effects by inhibiting or stimulating breast cancer at different dose [47]. What’s more, the studies in vivo have suggested that ZEA was involved in the increasing the risk of hormone-dependent tumors and might exert a crucial role in the increasing incidence of cancer in different organs [48,49]. After exposure to ZEA at an environmentally relevant doses, the female rat mammary gland was changed, which might increase the incidence of mammary tumors [50]. In addition, after feeding with dietary ZEA for 104 weeks, the weights of liver were increased in both male and female rat and the uterine were increased in females [51]. ZEA could also induce adverse liver lesions with subsequent development of hepatocarcinoma in B6C3F1 Mice and F344/N Rats [52]. The alteration and increasing in the weights of these organs is probably a precursor of the carcinogenic property.

#### The Mechanisms of ZEA Exhibited the Carcinogenic Property

Firstly, ZEA could cause DNA damage and induce chromosome aberrations, which might be an important reason for inducing cancer. Reports have suggested that ZEA was cytotoxic and genotoxic, and was able to induce DNA fragmentation and chromosome aberrations were statistically increased dose-dependently from 10 to 40 µM of ZEA [5,53]. The DNA lesions induced by ZEA were paired by unscheduled DNA synthesis (UDS). If the error occurred in the process of the DNA repair, the mutation can take place in DNA [53]. ZEA also induced SOS repair process in lysogenic bacteria and DNA-adduct formation in mouse tissues [54]. ZEA could also destroy the double strand of DNA, which could activate the DNA repair gene including RAD51 and BRCA1and the DNA damage repair systems [55]. Several studies have demonstrated that ZEA also can induce chromosomal polyploidy, aberrations and sister chromatid exchanges in the cells of Chinese hamster ovary [5,56]. ZEA exerted its chromosome-damaging effects in the nucleus through binding to ERs or steroid receptors [57]. The accumulation of mutations induced by oxidative damage is a possible contributor to mitochondrial dysfunction. Dysfunctional mitochondrion can increase the level of reactive oxygen species (ROS) which can cause the mutation. Thus, it may be a feed-forward loop where ROS destroy the mitochondria and the damage mitochondria can generate more ROS [58,59,60]. 

Secondly, many studies have revealed that ZEA could disturb the function of gap junctional intercellular communication (GJIC) [53]. It has reported that the dysfunctional GJIC was a precursor or prerequisite of the tumor. It seems to play a crucial or give a significant effect on the process of tumors especial for solid tumors. The invasion stage being mostly associated with a loss of function of the gap junctions [61]. GJIC played an important role of tumorigenesis and was a key mechanism in regulating physiological homeostasis. The protein Cx43 was transported from trans-Golgi network to cytomembrane through a microtubules [62]. ZEA can destroy the dynamic balance between cell growth and death and cause abnormal regulation of oncogenes. What’s more, ZEA could increase the expressions of proto-oncogenes including c-Myc, c-Fos\and c-Jun and could also decrease the expressions of anti-oncogenes including PTEN and P53 in TM3 cells, which may be beneficial for inducing the translation of normal cells into abnormal cells including tumor cells [8].

## 4. The Molecular Mechanisms of ZEA Caused Cell Death

### 4.1. ZEA Causes Cell Death through Disturbing the Distribution of Cell Cycle and the Expressions of Cell Cycle Regulating Proteins

Several studies have indicated that the percentage of cell distribution was disturbed by ZEA in different cells [19,46,63]. ZEA could significantly affect the distribution of cell cycle and cause the cell cycle arrest at G2/M phase in MTEC1 cells after treatment ZEA for 24 h and 48 h in vitro [64]. The study from TM4 cells suggested a same result that ZEA could inhibit the cell Proliferation and cause cell cycle arrest at the G2/M Phase after treatment with different concentrations (0.1, 1, 10, 20 and 30 μM) of ZEA for 24 h in vitro [19]. ZEA decreased the number of cells in the G0/G1 and increased the number of cells in the G2/M phase in Vero, Caco-2 and DOK cells after treatment ZEA concentrations of 10, 20 and 40 μM in vitro [46]. ZEA could markedly affect the cell cycle distribution of human Embryo Kidney (HEK) 293 cell and ZEA decreased the cell number in the S phase and arrested the cell cycle in the G2/M phase [15]. What’s more, ZEA not only disturbed the distribution of cell cycle but also affected the expression of cell cycle-regulated proteins including Cyclin-B1, CyclinD1, CDK2 and CDK4 in a dose-dependent manner, which was induced through ROS-ER stress-AMPK pathway in mouse Sertoli cells [19]. Other studies indicated that the reason why cell cycle is arrested in G0/G1 or G2/M is for repair the DNA damage to take place and DNA damage was proved that was associated with a G2/M phase delay or arrest [65]. In recently, several studies have indicated that ZEA could cause DNA damages which may be one of the important reasons that ZEA arrested the cell cycle [5,53,66]. The arrest of cell cycle progression provokes the arrest of DNA replication and consequently the inhibition of cell proliferation [67].

### 4.2. ZEA Caused Cell Death through Inducing Cell Oxidative Stress

**Figure 3:**
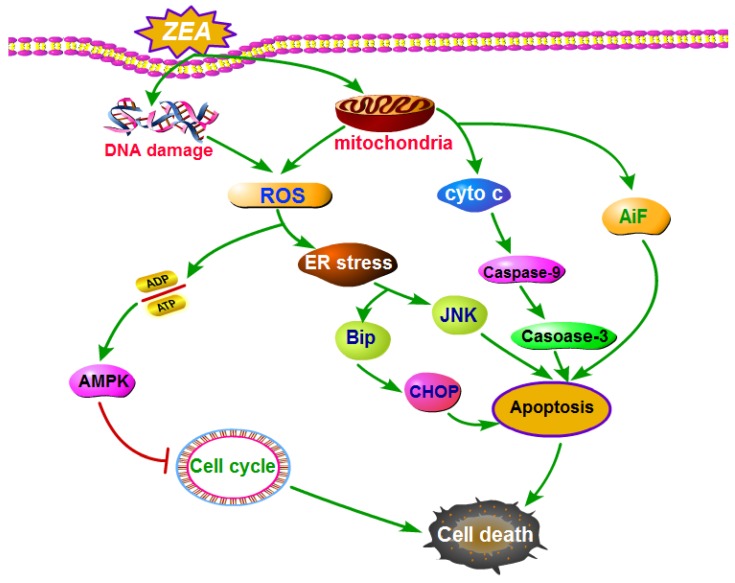
The molecule mechanisms of ZEA induced cell death. ZEA caused cell death through inducing cell apoptosis. ZEA induced cell apoptosis through ER stress, ROS and mitochondrial signaling pathway. Abbreviations: Zearalenone (ZEA); reactive oxygen species (ROS); AMP-activated protein kinase (AMPK); endoplasmic reticulum (ER) stress; Binding immunoglobulin protein (BiP); c-Jun amino terminal kinase (JNK); C/EBP homologous protein (CHOP); cytochrome c (cyto c); apoptosis inducing factor (AiF).

An increasing number of studies have revealed (Figure 3) that ZEA can cause oxidative stress and produce ROS and the overproduction of ROS participated in the process of cell death induced by ZEA [68,69]. ZEA could generate ROS and induce oxidative stress during the formation of its metabolites. ZEA and its derivatives such as α-ZEL and β-ZEL, resulted in damage of DNA and increased the level of ROS [70]. It was reported that ZEA was catalyzed into two main reductive metabolites including α–ZEL and β-ZEL by 3α-/3β-HSD [71,72]. A study has identified that 13-hydroxy-ZEA and 15-hydroxy ZEA were two highly unstable oxidative metabolites which were indicted to exert the potency of causing the oxidative DNA damage as catechols of estradiols, which was detected by the level of 8-oxo-2′-deoxyguanosine of DNA [73]. What’s more, the generating of ROS might be attributed to the alteration of mitochondrial NADPH-oxidase which can catalyze normal oxygen into superoxide anions [74]. After exposure to ZEA for 6 and 24 h, the expression of NADPH –oxidase was upregulated by 1.32 and 1.6 fold respectively [71].

In addition, it was known that the mitochondria are the largest contributors of producing intracellular ROS in most cell types [75]. Several enzymes were involved in the process of generating ROS in mitochondria and the nicotinamide adenine dinucleotide dehydrogenase exerted a crucial role in this process [76,77,78,79]. Accumulating evidence showed that the aberrant and excessive action of destroying normal proteins and organelles including mitochondria, which might generate ROS [80]. Several studies have suggested that ZEA altered the structure of mitochondrion and with the increasing doses of ZEA the disruption was more severe [14]. After treatment with different concentrations of ZEA the mitochondrial membrane potential was significantly decreased in a dose-dependent manner [14]. Therefore, ZEA generated ROS may also through damaging of mitochondrion and inducing mitochondrial membrane permeabilization and inactivation of cytochrome c oxidase. 

However, mitochondria are not only the major source of ROS but also the targets of cellular ROS [81]. High concentration of ZEA could induce imbalance of the oxidative system in IPEC-J2 cells and the accumulation of ROS by inadequate antioxidant defenses, which would aggravate the damage to the mitochondrial function [82].

There is a feed-forward vicious cycle between generating of ROS and damage of mitochondria in which the mitochondria damaged by ROS lead to dysfunction and the dysfunctional mitochondria could further cause the overproduction of intracellular ROS. When the mitochondria were involved in this feed-forward cycle, NADPH oxidase could not control the fate of cells. Thus, only the antioxidants, for example, MitoQ targeting mitochondria could destroy this feed-forward vicious cycle and saved the cells from death [75]. 

### 4.3. ZEA Caused Cell Death through Inducing Cell Apoptosis

In recent years, numerous studies have demonstrated that the cell death caused by ZEA was attributed to the cell apoptosis. ZEA treatment could induce cell apoptosis in different cell types. After exposure to ZEA (30 and 50 µM) for 12 h, the early apoptotic cells were detected, while after exposure to ZEA for 24 h, the late apoptotic were mainly observed in vitro [83]. ZEA significantly increased the apoptosis ration from 6.18% to 35.66%, and the mitochondrial membrane potential was decreased in a dose-dependent manner after treatment with ZEA (0.1, 1, 10, 20 and 30 µM) in TM4 cells in vitro [19]. ZEA resulted in an obvious cell apoptosis and increased the the expressions of cleaved caspase-3 and caspase-9 and the ratio of Bax/Bcl-2 [18]. After exposure to 15, 30, and 60 μM of ZEA for 48 h, the levels of PARP and AIF were increased and the level of BCL-2 was decreased in a dose-dependent manner in hTERT-GLCs in vitro [84]. Although there are a lot of signaling pathways which are involved in ZEA induced cell apoptosis, the main pathways are ER stress, oxidative stress and mitochondrial signaling pathways [85].

#### 4.3.1. ZEA Induced Cell Apoptosis through ER Stress

An increasing number of studies have suggested that ER stress pathway was revealed as an important pathway of ZEA induced cell apoptosis. ZEA can induce apoptosis in RAW 264.7 macrophages through the ER stress pathway [86]. ER stress induced cell apoptosis by activating pro-apoptotic molecules including transcriptional factor C/EBP homologous protein (CHOP), apoptosis signal-regulating kinase 1 (ASK1)/c-Jun amino terminal kinase (JNK), and caspase 12 [87]. The marker molecules of ER stress including BiP and CHOP were found to be up-regulated after treatment with ZEA in a time and dose-dependent manner and the increasing expressions of the marker molecules of ER stress could markedly be reduced by using the ER stress inhibitor 4-PBA. After using lentivirus-mediated shRNA to decrease the expression of CHOP, the rate of cell death and apoptosis was diminished [83]. Besides these traditional and classical pathways of apoptosis induced by ER stress, recent studies also found that the other signal ways including ATP/AMPK were also mediated by ER stress in the process of cell cycle arrest and apoptosis induced by ZEA [19]. 

#### 4.3.2. ZEA Induced Cell Apoptosis through Producing of ROS

It is known that accumulation of intracellular ROS can cause oxidative stress, which can lead to cell death through apoptosis [22]. ZEA can cause oxidative stress and stimulate the gradually increasing of ROS, Superoxide dismutase (SOD), Malondialdehyde (MDA) in a dose-dependent manner [88]. The overproduction of ROS was involved in the process of apoptosis induced by ZEA, and the rate of apoptosis was decreased significantly after co-treatment with NAC and ZEA [19]. The generation of ROS could stimulate the level of ER stress which can induce cell apoptosis. After using the NAC, the level of ER stress including several molecular indicators of ER stress including the expressions of Bip, CHOP, ATF6 and PERK and the level of intracellular Ca^2+^ were decreased by using NAC to alleviate the level of oxidative stress [89]. ZEA caused cell death including apoptosis and necrosis in RAW 264.7 cells through the AIF and ROS signaling pathways, and the process was regulated by the activation of p53 and MAPK signal ways [22].

#### 4.3.3. ZEA Induced Cell Apoptosis through Mitochondrial Signaling Pathway

In addition to the above mentioned pathways, the mitochondrion-initiated intrinsic pathway was also found to participate in the process of ZEA induced apoptosis [90]. There are two major pathways involved in the process of apoptotic cell death one is the mitochondrial pathway through releasing cytochrome c into cytosol and the another one is the death receptor pathway mediating by caspase-8 [90]. ZEA significantly inhibited the growth and caused cell death through inducing apoptosis. The cytochrome c in cytosol was increased and the caspase-9 and caspase-3 were activated after treatment with ZEA in Leydig cells [23]. ZEA could induce cell apoptosis in porcine granulosa cells through the mitochondrial and caspase-dependent pathway [18]. ZEA could also affect the expressions of Bcl-2 family proteins including up-regulating Bax and down-regulating Bcl-2 expression [90,91]. 

ZEA and its metabolites could cause the decreasing of mitochondrial membrane potential (MMP), and cytoplasmic release of cytochrome c in raw264.7 cells [89]. What’s more, ZEA could induce cell apoptosis through p53-dependent mitochondrial signaling pathway [92]. ZEA could induce cell apoptosis through activating or changing the expression p53 to regulate the p53-mediated apoptosis pathway [93]. P53 is a transcription factor that can repress or activate the expressions of multiple genes. The activating of p53 –dependent pathway can lead the changes of mitochondrial apoptotic including releasing cytochrome c and activating the caspase cascade through extrinsic and intrinsic signal ways to trigger the cell death [63].

The data from Table 1 has suggested that ZEA could stimulate cell proliferation in different cells at a low dose starting from 10^−11^ M. A low dose of ZEA can exert the estrogen-like effects and exhibit the carcinogenic property, which can stimulate the proliferation of cells. In the cells or tissues which could express the estrogen receptors or be regulated by estrogen such as T47D cells, MCF-7 cells, TM3 cells and uterine, ZEA might exhibit its estrogen-like effects to increase cell proliferation. While in the cells or tissues which have no estrogen receptors, such as Granulosa cells, Human colon carcinoma cells (HCT116), SK-N-SH human neuroblastoma cells and Liver, ZEA might exert its carcinogenic property to stimulate cell proliferation increase. The data from Table 1 has also shown that ZEA could cause cell death at a high dose starting from 10^−5^ M in different cell types including Porcine granulosa cells, Mouse Leydig cells, HEK293 cells, Mouse ES cells, PK15 cells, TM4 cells, T47D cells and MCF-7 cells. High dose of ZEA could cause the oxidative stress and generated ROS which can induce ER stress, apoptosis, and even DNA damage. Taken together, these summaries suggested that a low dose of ZEA stimulated cell proliferation through the estrogen-like effects and carcinogenic property and a high dose of ZEA caused cell death through inducing oxidative stress, DNA damage, mitochondrial damage, cell cycle arrest and apoptosis.

## 5. What Can Protect the Damage or Cell Death Induced by ZEA

### 5.1. ZEA Can Induce Autophagy Which Can Exert a Protective Function Against the Cytotoxicity

The cell could maintain a very low level of autophagy under the normal physiological conditions. While, when the cells were subjected to various stress conditions including nutrient limitation, endoplasmic reticulum stress and oxidative stress the autophagy will be triggered in order to survive [96]. Several reports have suggested that the cellular integrity and homeostasis were maintained by autophagy through damaging organelles and degrading cytosolic macromolecules in response to the stress or under normal physiological conditions [97,98]. Several studies have suggested that autophagy acted a crucial role in protecting the cells against cytotoxicity induced by ZEA [86]. Autophagy is a double-edged sword in the process of cell death and damage, but there is accumulating evidence that autophagy exerts a protective function against the cytotoxicity induced by ZEA. ZEA can activate autophagy and apoptosis in Leydig cells and autophagy delays apoptosis in primary rat Leydig cells [23]. Using the shHerp to reduce the expression of Herp could stimulate the level of autophagy, which exerted a protective role in ZEA-induced cell death [86]. The activating of autophagy could serve as a cardioprotective mechanism against these mycotoxins including and counteracting apoptosis [99].

Autophagy can be triggered by several interconnected signal pathways including MAPK family, PI3K-AKT-mTOR and ADP/ATP-AMPK signaling pathways [100]. Studies have revealed that the ERK and PI3K-Akt-mTOR signaling pathways participated in the process of autophagy induced by ZEA. The ERK signaling pathway triggered the autophagy in TM4 cells through suppressing the phosphorylation of mTOR [88]. What’s more, the increasing evidence has indicated that the ER stress pathway is another significant pathway to induce autophagy. With the accumulation of unfolded proteins and damaged organelles, the autophagy can be activated by them during ER stress [101]. ER stress signaling pathway was involved in the induction of autophagy in TM4 cells. ER stress was involved in the induction of autophagy via inhibiting the ERK signal way to suppress the phosphorylation of mTOR [88]. Additionally, the AIF-mediated pathway exerted an important role in ZEA-induced autophagy. Apoptosis inducing factor (AIF), a key caspase-independent death effector, is cleaved into its mature form and translocated to the nucleus from the mitochondria. The knockdown of AIF could promote the switch of LC3-I to LC3-II and increase the level of autophagy in goat Leydig cells [84]. 

Interestingly, several studies have also reported that deoxynivalenol (DON), another toxin produced by fusarium species often co-contaminated with ZEA, DON could inhibit plant apoptosis-like programmed cell death induced by heat shock and ethanol treatment in Arabidopsis cells at low dose [19]. The reason that DON could inhibit apoptosis-like programmed cell death might be also attributed to the protective function of autophagy. This is because DON could also cause trigger cell autophagy and apoptosis simultaneously in different cell types including human and animals [102,103,104]. What’s more, a study has shown that autophagy could provide protective function against the cytotoxicity induced by DON through reducing the level of ROS and maintaining the cellular stress response in the intestinal epithelial Cells [105]. Autophagy may play a critical role in the different cells against the cytotoxicty of the Fusarium mycotoxins.

### 5.2. Some Antioxidants Which Can Alleviate the Oxidative Damage May Partially Prevent the Cytotoxicity Induced by ZEA

ZEA could stimulate oxidative stress and produce ROS, which can cause cell death, induce cell apoptosis and exert cytotoxicity. Some antioxidants including Proanthocyanidin, NAC and Vitamin E were verified that could partially reverse the cytotoxicity induced by ZEA. Proanthocyanidin, one of the most abundant groups of natural polyphenols, was regarded as functional ingredients with anti-oxidant [106]. Studies have suggested that Proanthocyanidins could ameliorate testicular reproductive toxicity and decrease the level of cell apoptosis induced by ZEA reducing the oxidative stress and down-regulating the level of ER stress [107]. NAC, a well-known thiol antioxidant that acts as a glutathione precursor was widely used in clinical practice [108]. Studies have demonstrated that NAC could partially reverse the damages including oxidative damage and apoptosis induced by Fusarium toxin in PK15 cells [20]. What’s more, studies have showed that ZEA induced epigenetic effects and genotoxic including inhibiting GJIC, chromosome aberrations and DNA lesions through oxidative process, which was partially prevented by using Vitamin E [53,57].

## 6. Conclusions and Future Perspective

ZEA exerts different mechanisms of toxicity in different dose and cell types. A low dose of ZEA can exert the estrogen-like effects and exhibit the carcinogenic property, which can stimulate the proliferation of cells. While, a high dose of ZEA can cause cell death through inducing oxidative stress, DNA damage, mitochondrial damage, cell cycle arrest and apoptosis. In the cells from tissues which can express estrogen receptors including testes, ovary, mammary glands, uterus, vagina, epididymis, and prostate ZEA can exhibit the estrogen-like effects [109]. However, in the cells from the tissues which have no estrogen receptors, ZEA may exert the cytotoxicity such as the carcinogenic property, DNA damage, and oxidative stress.

A large number of studies have indicated that ZEA and its derivatives can exert estrogen-like effects and bind to estrogen receptors. Due to the different molecular structure, the estrogenic activities are different and can be ranked as follows: α-ZEL = α-ZAL > ZAN > ZEA = β-ZAL > β-ZEL. As a result, these derivatives should be taken into consideration when we are designing the related research to detect the cytotoxicity of ZEA, especially the estrogen-like effects. It is predicted that in the future, the related research about the combined toxicity of ZEA and its derivatives will become a trend. In addition, an increasing amount of data has suggested that ZEA can increase the level of ROS and cause damage to mitochondria and DNA. However, the relationship between ROS and the damage of mitochondria and DNA is controversial. Mitochondria are not only the major source of ROS but also the targets of cellular ROS. The overproduction of ROS can cause DNA damage, while the damage of DNA can cause the generation of ROS. Thus, it is recommended that the related research should be designed to explore the relationship between generating ROS and destroying mitochondria and DNA.

## Figures and Tables

**Table 1:** ZEA stimulated cell proliferation and caused cell death in different cell types.

Mycotoxin	Exposure Dose	Exposure Period	Cell Line or Tissues	Effects	Ref
ZEA	10^−10^–10^−8^ M	6 d	T47D cells	Cell proliferation 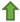	[10]
ZEA	10^−10^–10^−8^ M	6 d	MCF-7 cells	Cell proliferation 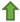	[10]
ZEA	10^−11^–10^−6^ M	24 h	KK-1 cells	Cell proliferation 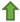	[2]
ZEA	10^−7^ M	36 h	MCF-7 cells	Cell proliferation 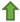	[94]
ZEA	10^−8^–10^−9^ M	7d	T47D cells	Cell proliferation 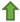	[11]
α-ZEL	10^−9^–10^−6^ M	7d	T47D cells	Cell proliferation 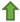	[11]
α-ZAL	10^−7^–10^−5^ M	5 d	BMS Cells	Cell proliferation 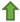	[12]
ZEA	10^−11^–10^−5^ M	72h	MCF-7 cells	Cell proliferation 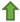	[36]
ZEA	10^−8^–10^−5^ M	6d	MCF-7 cells, T47D cells,	Cell proliferation 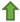	[10]
ZEA, FB1	ZEA 9.4 µM	48 h	Granulosa cells	Cell proliferation 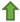	[13]
	FB1 3.4 µM				
ZEA	10^−9^–10^−6^ M	0-120 h	HCT116	Cell proliferation 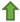	[45]
ZEA	10^−8^ M	72 h	TM3 cells	Cell proliferation 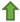	[8]
				Cell apoptosis 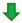	
ZEA	10^−8^–10^−6^	72 h	SK-N-SH cells	Cell proliferation 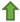	[95]
ZEA	0.1, 1.0 and 10.0 mg per kg body weight per day	10 months	Liver, uterine	The weights of liver and uterine 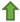	[51]
ZEA	60, 90, and 120 µM	24 h	Porcine granulosa cells	Cell apoptosis 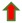	[18]
ZEA	0–600 µM	24 h	Mouse Leydig cells	Cell apoptosis 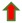	[22]
ZEA	50–300 µM	24 h	HEK293 cells	Cell viability 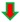	[15]
ZEA	25, 50, 75, and 100 μM	24 h	Mouse ES cells	Cell apoptosis 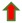	[21]
ZEA	20 μM	24 h	PK15 cells	Cell viability 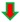	[20]
ZEA	1, 10, 20, 40, 60, 80 and 100 μM	24 h	TM4 cells	Cell apoptosis 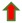	[19]
				Cell viability 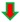	
ZEA	15–60 μM	24 h	Sertoli cells	Cell death 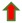	[14]
				Cell viability 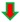	
ZEA	3–300 μM	24 h	HEK 293 cells	Cell apoptosisthe 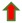	[15]
				cell viability 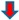	
ZEA	10, 20 and 40 μM	24 h	Caco-2, DOK and Verocells	Cell apoptosis 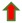	[46]
ZEA	10^−5^ M	6 d	T47D cells	Cell death 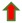	[10]
ZEA	10^−5^ M	6 d	MCF-7 cells	Cell death 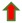	[10]
ZEA	10^−6^–10^−4^ M	24 h	KK-1 cells	Cell death 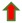	[2]
